# The Sustained Attention to Response Task Shows Lower Cingulo-Opercular and Frontoparietal Activity in People with Narcolepsy Type 1: An fMRI Study on the Neural Regulation of Attention

**DOI:** 10.3390/brainsci10070419

**Published:** 2020-07-01

**Authors:** Jari K. Gool, Ysbrand D. van der Werf, Gert Jan Lammers, Rolf Fronczek

**Affiliations:** 1 Department of Anatomy and Neurosciences, Amsterdam UMC, Location VUmc, De Boelelaan 1108, 1081HZ Amsterdam, Noord-Holland, The Netherlands; yd.vanderwerf@amsterdamumc.nl; 2 Sleep-Wake Centre SEIN, Achterweg 2, 2103SW Heemstede, Noord-Holland, The Netherlands; g.j.lammers@lumc.nl (G.J.L.); r.fronczek@lumc.nl (R.F.); 3 Department of Neurology, Leiden University Medical Centre, Albinusdreef 2, 2333ZA Leiden, The Netherlands

**Keywords:** disorders of excessive somnolence, narcolepsy, hypocretin, magnetic resonance imaging, sustained attention to response task, vigilance

## Abstract

Vigilance complaints often occur in people with narcolepsy type 1 and severely impair effective daytime functioning. We tested the feasibility of a three-level sustained attention to response task (SART) paradigm within a magnetic resonance imaging (MRI) environment to understand brain architecture underlying vigilance regulation in individuals with narcolepsy type 1. Twelve medication-free people with narcolepsy type 1 and 11 matched controls were included. The SART included four repetitions of a baseline block and two difficulty levels requiring moderate and high vigilance. Outcome measures were between and within-group performance indices on error rates and reaction times, and functional MRI (fMRI) parameters: mean activity during the task and between-group activity differences across the three conditions and related to changes in activation over time (time-on-task) and error-related activity. Patients—but not controls—made significantly more mistakes with increasing difficulty. The modified SART is a feasible MRI vigilance task showing similar task-positive brain activity in both groups within the cingulo-opercular, frontoparietal, arousal, motor, and visual networks. During blocks of higher vigilance demand, patients had significantly lower activation in these regions than controls. Patients had lower error-related activity in the left pre- and postcentral gyrus. The time-on-task activity differences between groups suggest that those with narcolepsy are insufficiently capable of activating attention- and arousal-related regions when transitioning from attention initiation to stable attention, specifically when vigilance demand is high. They also show lower inhibitory motor activity in relation to errors, suggesting impaired executive functioning.

## 1. Introduction

Narcolepsy type 1 is a severely disabling neurological condition caused by a selective loss of hypocretin-producing neurons in the lateral and posterior hypothalamus [1,2]. It is characterized by excessive daytime sleepiness (EDS), cataplexy, sleep paralysis, hypnagogic hallucinations and disturbed nocturnal sleep. People with narcolepsy type 1 also frequently report vigilance (also called “sustained attention” or “tonic alertness”) deficits, considered to be a result of EDS [3,4,5]. The ability to remain vigilant is essential for effective daytime functioning [6,7]. People with narcolepsy therefore often report difficulties with studying or working, and are more frequently involved in traffic accidents [8,9,10]. Here, we report on a functional MRI (fMRI) study investigating the neurobiological basis of the vigilance difficulties in people with narcolepsy type 1 using the sustained attention to response task (SART). The neural correlates of vigilance regulation in general, different stages of attention and response inhibition capacities were studied.

Various, often lengthy, tasks have been used to investigate vigilance in people with narcolepsy. First described by Robertson et al. (1997), the SART is a relatively short “Go/No-Go” task requiring continuous decision making depending on the repetitive presentation of visual stimuli. These stimuli include single digits (1–9) that are transiently presented in a pseudorandom order with relatively sparse No-Go trials. The participant is instructed to respond with a button press, unless a “3” is being shown. Performing the task requires both sustained attention and executive functioning (including response inhibition) and has previously been validated for assessing everyday cognitive slips in people with narcolepsy and healthy controls [11,12,13,14]. Previous behavioral SART research in people with narcolepsy type 1 has revealed significantly worse test performance and longer reaction times than in healthy controls [11,13,14]. No study has yet focused on the neural substrates of attention regulation in narcolepsy type 1.

Variable SART paradigms have been used in an MRI environment in healthy individuals, mainly differing in length and the makeup of the baseline condition. There is evidence that the SART induces task-positive cingulo-opercular, frontoparietal, and supplementary motor cortex activation in healthy participants [15,16,17,18]. However, it remains unknown how changes in vigilance demand, different stages of attention and errors are processed in the brain. 

In this study, we tested the feasibility of a multilevel SART paradigm with increasing vigilance demand over two difficulty levels of moderate and higher difficulty in healthy participants and in individuals with narcolepsy type 1. We identified the behavioral performance indices and neural correlates of varying vigilance need and of changes over time (time-on-task effects) reflecting different stages of attention in people with narcolepsy type 1 and healthy controls. Error-related brain activity was also compared between groups by adding participants’ error scores as covariates of interest to the analyses of the main effects, i.e., task vs. baseline. We hypothesized that people with narcolepsy type 1 would show lower activity than controls in the cingulo-opercular and arousal networks when performing the task, particularly in the higher difficulty level. People with narcolepsy were also expected to have lower activity within these networks when progressing the task. 

## 2. Materials and Methods

### 2.1. Participants

Twelve adults with narcolepsy type 1 were recruited through the outpatient clinic of Sleep-Wake Centre SEIN (The Netherlands) and twelve age and sex group-matched healthy participants were recruited through local newspaper advertisements. People with narcolepsy type 1 were diagnosed according to the 3rd edition of the International Classification of Sleep Disorders (ICSD-3) [19]. All participants had to be 18-65 years old, right-handed and have normal or corrected-to-normal vision. Those with narcolepsy type 1 needed to be treatment-naïve or treatment-free for at least two weeks before MRI acquisition. Exclusion criteria consisted of current psychotropic drug usage, present diagnosis of a serious comorbidity, contraindications for MRI scanning and macroscopic structural brain abnormalities (tumors, ventricle enlargement, cortical atrophy, or vascular lesions). As described in the ICSD-3 diagnostic criteria, the cerebrospinal fluid (CSF) hypocretin-1 levels of the three people with narcolepsy type 1 without clear-cut cataplexy were determined. Notably, one patient had a concentration (138 pg/mL) slightly above the threshold according to international standards of 110 pg/mL for narcolepsy type 1 [20]. This individual was not excluded because the clinical phenotype was typical and the hypocretin-1 level still deficient. The same population of participants was previously studied using diffusion tensor imaging to analyze microstructural white matter integrity [21].

Before image acquisition, the Dutch version of the National Adult Reading Test [22] was administered to assess intelligence (IQ) and the Epworth Sleepiness Scale (ESS) was used to measure daytime sleepiness [23]. All data collection was performed during the afternoon to account for possible circadian effects. Participants were asked to refrain from caffeine-containing substances for at least 24 hours prior to examination. All provided written informed consent prior to study start. The study was approved by the Medical Ethical Committee of Leiden University Medical Center (LUMC), The Netherlands (NL46982.058.14) and was conducted according to the Declaration of Helsinki.

### 2.2. Sustained Attention to Response Task

**Figure 1:**
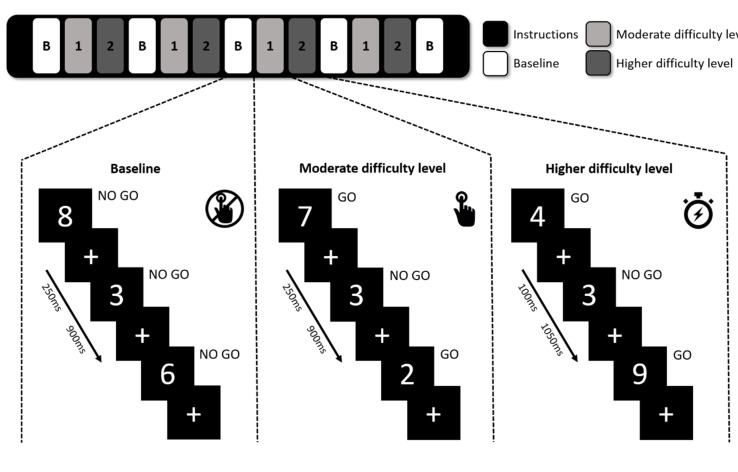
The modified sustained attention to response task paradigm. In baseline blocks, participants did not have to respond (No-Go). In the moderate and higher difficulty level participants had to responded by button press to all digits (Go) except for the digit “3” (No-Go). The difficulty levels differed in duration of stimulus presentation (250 ms in moderate difficulty level and 100 ms in higher difficulty level).

During the regular SART, participants are shown single digits from 0 to 9 for 4.19 min and are asked to press a key when a digit appears (GO), unless the digit is a “3” (No-Go). As shown in Figure 1, a modified version of the SART was used in this study to identify the participants’ ability to adapt to changes in vigilance demand by introducing a baseline condition and two difficulty levels of moderate and higher difficulty. In the baseline block, participants were instructed to focus on the appearing digits-including 3s-without pressing the button, while in moderate and higher difficulty levels they were instructed to respond to non-3 stimuli by pressing a button using their right index finger. Each block consisted of 27 visual stimuli and in the moderate difficulty level the digits were visible for 250 ms compared to just 100 ms in the higher difficulty level, to create a state of higher vigilance demand. A fixation cross was presented after the digit to make sure the participant had enough time to respond, and each stimulus-cross combination lasted for 1150 ms in total. A short instruction screen was shown when transitioning between different conditions. Our test consisted of four consecutive repetitions of the baseline, moderate difficulty, and higher difficulty blocks, ending with an extra baseline block and altogether with instructions lasted 9 min. Participants were instructed to focus equally on accuracy and speed while performing the task. The task was explained and briefly practiced before entering the MRI scanner.

The SART error scores were calculated separately for the two difficulty levels and compared between the groups as a measure of task performance. Mean reaction times (RTs) were determined per difficulty level relative to stimulus onset and compared between the groups. Additional mean error rates were calculated per difficulty level per repetition to assess the behavioral time-on-task effect over the entire SART. Mean scores were also computed separately for the early half and late half of each difficulty level to identify possible within-block time-on-task differences.

### 2.3. Image Acquisition

A high-field 3T Philips Achieva MRI scanner (Philips Healthcare, Best, The Netherlands) was used for MRI data acquisition with a 32-channel head coil and sponge cushions to minimize head movements. For functional MRI scans, whole-brain functional T2*-weighted MRI data were acquired using a gradient-echo planar imaging (EPI) sequence (38 slices with a 0.25 mm gap; repetition time (TR) = 2250 ms; echo time (TE) = 29.94 ms; field of view (FOV) 200 × 200 × 104.25 mm; matrix size 80 × 80; flip angle = 80°; 2.5 × 2.5 × 2.5 mm^3^ voxel size). For coregistration purposes, we also acquired T1-weighted MR images (220 slices; TR 8.2 ms; TE 3.8 ms; inversion time 670.4 ms; FOV 240 × 240 × 220 mm; matrix size 240 × 240; flip angle 8°; 1 × 1 × 1 mm^3^ voxel size).

### 2.4. MRI Processing

The fMRI images were preprocessed and analyzed using FMRIB’s Software Library (FSL FEAT, version 5.1, Oxford, United Kingdom). In brief, the scans were motion corrected, brain extracted, normalized, filtered using a 100 s high-pass filter, smoothed with a 5 mm full-width at half maximum (FWHM) Gaussian kernel and coregistered to the corresponding skull-stripped T1 image.

### 2.5. Main Task Effect

The main task effect was assessed as the BOLD activity difference combining the blocks of the two difficulty levels over all four repetitions compared to the baseline blocks. Each combined task block lasted for 62.150 seconds and the baseline blocks and individual difficulty levels for 31.075 seconds. Three first-level contrast images were created for each participant: task > baseline; baseline > task; higher difficulty level > moderate difficulty level. All higher-level main task effect analyses results were masked inclusively for the (sub) cortical grey matter, brainstem, and cerebellum. As visual inspection of second-level comparisons on task effect showed similar activation patterns in both groups and no significant between-group differences were seen in the formal group comparisons, we pooled the corresponding contrasts for healthy controls and people with narcolepsy type 1 in one group to increase power of the main task effect.

### 2.6. Time-On-Task Effect

To assess changes in brain activation over the entire SART, we compared task activation in the moderate and higher difficulty level combined between repetition one and four (62.150 seconds each) using the preceding baseline blocks as reference (31.075 seconds). In the within-block time-on-task effect, each block was evenly divided in two halves (15.538 seconds each) to reflect the transition from attention initiation to stable attention. 

A three-level procedure was used to study the overall time-on-task effect. In the first-level analysis, two contrasts were created per participant: task repetition 1 > baseline repetition 1; task repetition 4 > baseline repetition 4. These contrasts were fed in second-level analyses using fixed effects to generate within-participant contrasts bidirectionally comparing activation between repetition 1 and 4. Second-level contrasts were used for third-level analyses to assess the overall time-on-task effect per group and to compare both groups.

The within blocks time-on-task effect was studied combining the two difficulty levels by creating two first-level contrasts: early half block > late half block; late half block > early half block. These contrasts were analyzed per group and were compared between groups in second-level analyses. Post-hoc analyses were conducted by separating the moderate and higher difficulty levels as significant differences were seen between groups within blocks with the two difficulty levels combined. 

### 2.7. Error-Related Effect

Block design analyses were implemented to compare error-related brain activity between people with narcolepsy type 1 and healthy controls as an event-related approach was not justified due to insufficient occurrence of errors. Error rates were calculated per participant for the two difficulty levels combined. Scores were added as covariates of interest in the contrast: task > baseline. Resulting contrasts were compared between the groups. 

### 2.8. Statistical Analyses

Statistical processing of demographic and task performance measures was done in IBM SPSS Statistics version 24 (International Business Machines Corporation, Armonk, NY, USA), with *p*-values < 0.05 considered statistically significant. Student’s *t*-tests were used for age, IQ, ESS, and behavioral SART performance comparisons between groups and a chi-squared test to identify possible sex differences. Overall SART, time-on-task over repetitions, and time-on-task within blocks performance measures were analyzed using repeated measures ANOVAs. Additional paired-samples *t*-tests were conducted on overall SART performance to analyze within-participant effects per group. Student’s *t*-tests were used to study differences per difficulty level. Bonferroni correction for multiple comparisons thresholds were calculated using Simple Interactive Statistical Analysis (SISA) separately for error scores (correlation = 0.65, N = 9, *p* < 0.024) and reaction times (correlation = 0.86, N = 9, *p* < 0.037). 

The main task, time-on-task and error-related fMRI effects were determined per group using one-sample *t*-tests. For group comparisons, two-sample *t*-tests were used. The main task effect analyses per group were controlled for type I errors using family-wise error (FWE) correction. Cluster-correction for multiple comparisons was used for main task effect group comparisons and all time-on-task and error-related fMRI analyses. A *p* < 0.05 threshold and minimum significant clusters size of 20 voxels were used. If time-series plots generated by FSL showed possible outliers, the corresponding analysis was repeated with automatic outlier deweighting. The exact location of significant clusters was determined using the AAL and Brodmann+ atlases in the WFU PickAtlas toolbox (Wake Forest University, Winston-Salem, NC, USA, version 3) as part of SPM 12.

## 3. Results

### 3.1. Demographic and Clinical Data

Data of 12 people with narcolepsy type 1 and 11 healthy controls were included as MRI scan acquisition failed in one healthy control. Both groups were comparable in age, IQ, and sex distribution (Table 1). Typical narcolepsy-related clinical measures were seen in those with narcolepsy type 1, including a significantly higher ESS score than controls (10.08 ± 3.00 vs. 2.64 ± 1.96; t(21) = −6.97, *p* < 0.001). All participants with narcolepsy type 1 were HLA DQB1*06-02 positive.

### 3.2. Behavioral Effects

**Figure 2:**
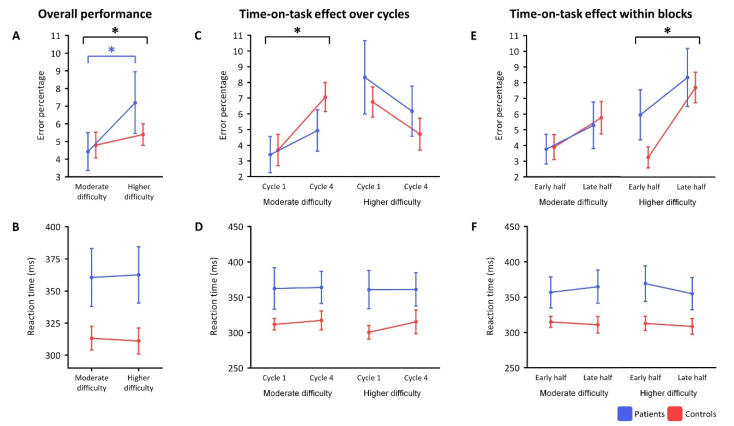
Behavioral results. (**A**,**B**) show the performance over the entire task per group. (**C**,**D**) show the time-on-task performance over repetitions (repetition 1 vs. repetition 4). (**E**,**F**) show the time-on-task performance within blocks (early half vs. late half). Significant differences (*p* < 0.05) are highlighted with an asterisk (*) and significant differences in black represent all subjects combined.

Behavioral analyses are visualized in Figure 2. For a complete overview of behavioral SART analyses, see Supplementary Material 1.

Two-way ANOVA showed a significant main effect for the difficulty level (F(1,21) = 9.04, *p* = 0.007), such that the average error score over all participants was significantly larger in the higher than the moderate difficulty level (Figure 2A). When splitting the groups, people with narcolepsy type 1—but not controls—made significantly more mistakes upon increasing difficulty (t(11) = −3.07, *p* = 0.011). Reaction times were numerically, but not significantly, longer in people with narcolepsy type 1 than in controls over the two difficulty levels (F(1,21) = 4.05, *p* = 0.057) (Figure 2B). 

Over repetitions, significantly more mistakes were made in repetition 4 than repetition 1 in the moderate difficulty level (F(1,21) = 11.88, *p* = 0.002), but not the higher difficulty level (Figure 2C). This increase in error scores in the moderate difficulty level was not significantly different between participant groups. No significant main effects on reaction time were found in the time-on-task analyses over repetitions (Figure 2D).

The time-on-task within blocks analysis showed that more mistakes were made in the late half compared to the early half of the blocks of the higher difficulty level when combining all participants (F(1,21) = 25.37, *p* < 0.001) (Figure 2E). This increase in errors in the higher difficulty level did not significantly differ between people with narcolepsy type 1 and controls (F(1,21) = 2.32, *p* = 0.143). No significant main effect of block half was found on mean error rates in the moderate difficulty level after correction for multiple comparisons (F(1,21) = 4.98, *p* = 0.037) or on the mean reaction times in both difficulty levels (Figure 2F).

### 3.3. Main Task Effects

**Figure 3:**
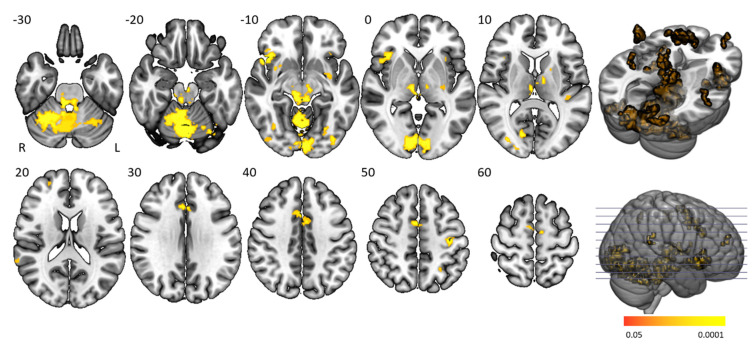
Main task effect activation clusters. Axial slices displaying significantly activated voxels in the “task > baseline” contrast over all subjects. Analyses were family-wise error (FWE)-corrected (*p* < 0.05), masked for grey matter, and a minimum cluster size > 20 voxels was used.

The “task > baseline” contrast elicited significantly stronger BOLD activation in the cingulo-opercular network, arousal system, motor (regulatory) areas, and visual cortex (Figure 3 and Table 2). The cingulo-opercular network was activated by means of the bilateral insula, thalamus, anterior cingulate cortex, and the right middle frontal gyrus. Other attention-regulation-related areas included the bilateral midcingulate cortex, right intraparietal sulcus and left inferior parietal gyrus being part of the frontoparietal network, vermis and bilateral inferior orbitofrontal gyrus, and fusiform gyrus. Motor (control) areas comprised the left pre- and postcentral gyrus, bilateral supplementary motor area, putamen, and cerebellum. The bilateral red nucleus, substantia nigra, and locus coeruleus within the midbrain and pons were also activated, with the pons being fundamental in managing arousal and attention. Task activation patterns of healthy controls and people with narcolepsy type 1 were similar and no significant main task effect differences were present between groups. No significant group differences were seen on the “baseline > task” and “higher difficulty level > moderate difficulty level” contrasts.

### 3.4. Time-On-Task Effects

**Figure 4:**
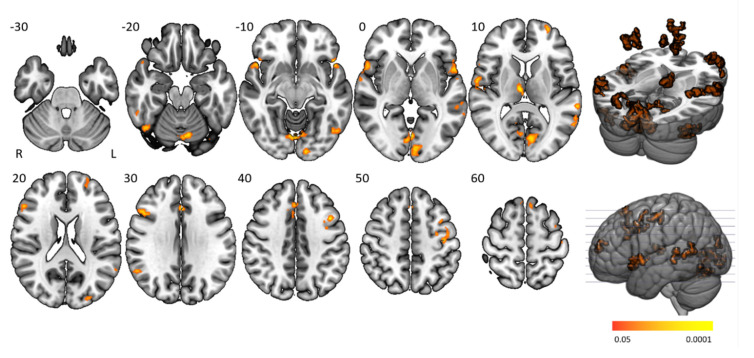
Time-on-task activation clusters within higher difficulty level blocks. Axial slices displaying significant activation clusters that were less activated in people with narcolepsy type 1 than in controls in the “late half > early half” contrast of the higher difficulty level. Analyses were cluster-corrected (*p* < 0.05), masked for grey matter and a minimum cluster size > 20 voxels was used.

Time-on-task analyses comparing BOLD signals in repetition 1 and 4 between participant groups yielded no significant activation differences. Time-on-task within blocks analyses demonstrated that people with narcolepsy type 1 had significantly less activation than controls in the “late half > early half” contrast combining the two difficulty levels. Post-hoc analyses showed that this effect was driven by the higher difficulty level, as shown in Figure 4 and Table 3. Significantly lower activation was found in people with narcolepsy type 1 in regions including the cingulo-opercular network (bilateral insula and operculum, left anterior cingulate cortex, right middle frontal gyrus, and thalamus), frontoparietal network (bilateral superior frontal gyrus, midcingulate cortex and right inferior frontal gyrus, and angular gyrus), (regulatory) motor areas, and visual regions. Other activated regions were the bilateral temporal lobe and inferior orbitofrontal gyrus and the right angular gyrus. The between-group differences were mainly driven by controls upregulating their neural efforts within higher difficulty blocks, whereas people with narcolepsy type 1 maintained stable activation over time. Decreased activation over time was only seen in those with narcolepsy type 1, in the visual cortex and cerebellum. No between-group time-on-task within blocks differences were seen in difficulty level 1.

### 3.5. Error-Related Effect

**Figure 5:**
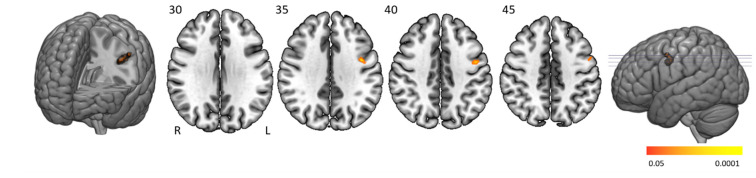
Error-related activation clusters. Axial slices displaying significant activation clusters that were less activated in people with narcolepsy type 1 than in controls in relation to error scores. Analyses were cluster-corrected (*p* < 0.05), masked for grey matter, and a minimum cluster size > 20 voxels was used.

People with narcolepsy type 1 had significantly lower activation than controls in the left pre- and postcentral cortex when making more errors (Figure 5 and Table 4). No significantly higher activation was found in people with narcolepsy type 1 compared to controls.

## 4. Discussion

The modified SART is a feasible MRI vigilance task showing similar cingulo-opercular, frontoparietal, arousal, visual, and motor-related activity in people with narcolepsy and controls. The same vigilance networks were activated in both groups when analyzing the main task and time-on-task effect over repetitions. When comparing activity in the first and second half of the higher difficulty level blocks, people with narcolepsy type 1 had significantly lower activation in the task-positive regions than controls. People with narcolepsy type 1 had significantly lower activation in the left pre- and postcentral cortex when making more errors, than healthy controls.

### 4.1. Behavioral Effects

Participants in both groups reported that the test was straightforward and easy to complete. Only individuals with narcolepsy type 1 made significantly more mistakes when difficulty increased and, in general, reacted more slowly than healthy controls. As well as intrinsic narcolepsy-related complaints resulting in longer response times, those with narcolepsy type 1 probably also sacrificed speed to improve accuracy at moderate vigilance demand (moderate difficulty level). They seemed unable to utilize this trade-off further when vigilance demand increased (higher difficulty level), resulting in an increase in mistakes in people with narcolepsy type 1, whereas healthy controls maintained a relatively stable performance. A similar speed–accuracy compensatory mechanism has previously been proposed by Van Schie et al. [13]. A possible learning effect was only seen when comparing performance during cycle 1 and 4 in the higher difficulty level, suggesting that participants at first were challenged more by the rapid stimulus presentation of the higher difficulty level than the moderate difficulty level.

### 4.2. Functional MRI Effects

Significant “task > baseline” activation was seen in the cingulo-opercular and frontoparietal attention network. Given the relative simplicity of the presented stimuli and the fast pace of stimuli presentation, we propose that task-positive activation of the cingulo-opercular network is related to enabling stable alertness and responsiveness throughout the entire task [24]. A similar role has been proposed for the cingulo-opercular activity as the core of task-set maintenance, related to sustaining attention and handling error processing [25,26]. The frontoparietal network on the other hand is involved in flexible adaptive control akin to its role in making rapid adjustments after stimulus presentation. This network is also implicated in attention initiation and error-related and visual information processing, all of which are fundamental SART domains [25,27,28,29,30,31]. The task-positive activation of the locus coeruleus is related to the increased state of arousal needed to complete the task [32].

The task-positive motor activation most likely reflects a combination of motor action through button presses and its preparation and inhibition (when a 3 was presented). Previous research has shown that performance in motor-related vigilance tasks, such as the SART, depends on preparatory (motor) set [33,34,35,36]. In our study we observed significant task-positive activity in multiple motor regions that are considered to control the Bereitschaftspotential (readiness potential) as they continuously assess both the planning of potential motor responses and prevent false premature initiation by limiting activity in nonprimary motor cortical areas through the thalamus and basal ganglia [37,38]. Notably, in this study, the left thalamus and subthalamic nucleus were also active while performing the task. 

Previous MRI-SART research only involving healthy participants found clear cingulo-opercular, frontoparietal and supplementary motor cortex activation, consistent with current understanding of attention systems in the brain [15,17,18]. These studies used a slightly different paradigm, including a baseline condition in which participants were instructed to keep responding to stimuli and a task condition similar to our moderate difficulty level [15]. Their main task effect is similar to ours, but generally smaller, as no significant clusters were reported in the cerebellum, thalamus, or basal ganglia. Discrepancies with our main task effect likely result from our different baseline condition in which no button presses were needed and our implementation of the higher difficulty level, which was substantially more difficult. 

Our results overlap with a substantial quantitative meta-analysis of 67 neuroimaging studies on different vigilance tasks, showing 11 neural clusters involved in vigilance regulation. In that analysis, significant activation was reported in the prefrontal cortex, anterior insula, parietal areas (intraparietal sulcus, temporo-parietal junction), and subcortical structures (cerebellar vermis, thalamus, putamen, midbrain). Similar to our study, a relative right lateralization of activation clusters was seen, which has been hypothesized as being related to maintaining stable attentional focus and response inhibition in studies using a similar Go/No-Go paradigm [33]. 

It seems contradictory that, despite the clear behavioral differences, the between-group and task-effect contrasts comparing both difficulty levels did not reach significance. This suggests that the neural differences are subtler than we hypothesized. Significant activation differences may be absent in the higher difficulty > moderate difficulty contrast in patients, but the corresponding behavioral abnormalities suggest future studies with increased power to demonstrate a significant neural effect. Larger participant groups are probably needed to be able to translate more behavioral results in differences in neural activation. No previous vigilance-related MRI studies in people with narcolepsy have been reported, making direct comparisons of our results impossible.

### 4.3. Time-On-Task Effects

Participants made significantly more mistakes in repetition 1 vs. repetition 4 in the moderate difficulty level. Interestingly, no between-group differences were found in the corresponding fMRI contrast. This could be related to the behavioral markers being more sensitive to short interruptions of instructions that were displayed between conditions, allowing the participants to recuperate. 

However, both time-on-task behavioral and fMRI differences were found comparing the two block halves of the higher difficulty level between groups. Controls, but not people with narcolepsy type 1, were able to activate further their task-positive networks when transitioning from the early half into the late half. Interestingly this is only found when vigilance demand is high. We suggest that maximal vigilance capacities have already been reached by people with narcolepsy type 1 in the early stage of the higher difficulty level, whereas controls are able to upregulate their cognitive effort to remain vigilant throughout the second half. It seems that people with narcolepsy type 1 experience problems transitioning from attention initiation to stable levels of attention. Interestingly, a similar stabilizing role has been proposed for the cingulo-opercular network during sleep, where its activity is positively correlated with stabilizing and deepening sleep through synchronization of the cyclic alternating pattern [39,40,41]. The question arises whether instability of the cingulo-opercular network as observed in our study during wake, could also play a role in disturbed sleep, potentially resulting in typical narcolepsy type 1 characteristics such as sleep state instability and fragmentation of nocturnal sleep. 

No studies have been published performing SART time-on-task analyses in people with narcolepsy, but previous clinical studies have reported similar cognitive difficulties in relation to lower vigilance capacities in people with narcolepsy [42]. The inability to sustain attention within blocks with high vigilance demand could resemble difficulties in daily life (e.g., studying, driving, working) experienced by people with narcolepsy type 1 [43]. 

### 4.4. Error-Related Effects

People with narcolepsy type 1 had significantly lower activation than controls in the left pre- and postcentral gyrus when making more errors. The primary motor and primary somatosensory motor cortex are known to be involved in motor response inhibition [44,45] and activation in these regions seems reasonable as most errors were errors of commission where the button should not have been pressed. Previous studies have shown that people with narcolepsy in general experience difficulties in impulsivity and response inhibition, specifically under time pressure [46,47]. Our results could therefore reflect the motor preparation-related aspect of these cognitive problems.

### 4.5. Study Limitations

All people with narcolepsy type 1 underwent extensive diagnostic testing, including genetic screening, and discontinued medication prior to study start to ensure homogeneity of the group, but it should be noted that this study only included relatively small participant groups. Future studies should include larger participant groups. 

In the traditional SART a monotonous task is performed for 4.19 min without interruptions. We needed to design the experiment in a way that conformed to limitations of the method, i.e., MRI-related signal drift. We therefore frequently alternated baseline and task conditions. This substantially limits the possibility of measuring continuous episodes of vigilance. Different neuroimaging modalities, such as electroencephalography (EEG) or magnetoencephalography (MEG), that are not susceptible to signal drift, could possibly be employed in future research.

We were unable to perform event-related analyses on error-related processing as a result of sparsity of errors. We suggest, therefore, that the task is extended and that only the higher difficulty level is used to increase the number of errors.

## 5. Conclusions

The modified version of the SART with varying levels of vigilance demand is a feasible MRI vigilance task for use in both people with narcolepsy type 1 and in healthy controls. Being vigilant during the performance of the SART resulted in activation of neural attention, motor (control), arousal, and visual networks. Even though people with narcolepsy type 1 made significantly more mistakes with increasing difficulty compared to relative stable performance in healthy controls, similar vigilance networks were activated during the task. Within higher difficulty level blocks, people with narcolepsy type 1 were less able than controls to activate task-positive networks over time. This possibly reflects their difficulties in transitioning from attention initiation to stable attention levels, specifically when vigilance demand is high. Lower motor-related activity was also seen in people with narcolepsy in relation to making errors, associated with impaired response inhibition. Better knowledge of vigilance-related networks will contribute to a better understanding of narcolepsy and other disorders of sleep and wakefulness, and possibly underlie more tailored approaches to therapeutic, medicated or nonmedicated, improvement of vigilance complaints in narcolepsy.

## Figures and Tables

**Table 1:** Characteristics of the study population.

	Patients (*n* = 12)	Healthy Controls (*n* = 11)	*p*-Value
Male:female (N:N)	8:4	7:4	0.879
Age (years, mean, SD)	33.25 (10.50)	31.82 (13.39)	0.777
IQ score (mean, SD)	110.58 (10.73)	111.30 (8.25)	0.865
Age of onset EDS (years, mean, SD)	19.42 (9.15)	-	
EDS duration (years, median, IQR)	10.00 (6.00-25.25)	-	
Cataplexy presence	9/12	-	
Cataplexy and/or hypocretin deficiency (N, %)	12/12	-	
HLA DQB1*0602 presence (N, %)	12/12	-	
ESS score (mean, SD)	10.08 (3.00)	2.64 (1.96)	<0.001
MSLT:			
Sleep latency (minutes, mean, SD)	4.62 (3.64)	-	
SOREM periods (mean, SD)	2.58 (1.57)	-	

EDS, excessive daytime sleepiness; HLA, human leukocyte antigen; ESS, Epworth sleepiness scale; MSLT, multiple sleep latency test; SOREM, sleep-onset rapid eye movement.

**Table 2:** Main task effect activation clusters.

Contrast	Anatomical Regions (AAL Atlas)	Anatomical Regions (Brodmann+ Atlas)	Cluster Size	Peak Z-Value	*p*-Value (FWE)	x, y, z
Task > Baseline	Bilateral cerebellum 4–6 Bilateral vermis 3–8, 10 Bilateral crus cerebelli 1 Bilateral calcarine cortex Bilateral lingual gyrus Thalamus (R)	Bilateral area 17 and 18 Midbrain (bilateral red nucleus, substantia nigra) Pons (bilateral locus coeruleus) Dentate nucleus (R) Medial dorsal nucleus (R)	4877	9.98	<0.0005	22, −62, −22
	Insula (R) Inferior orbitofrontal gyrus (R)	Area 47 (R), 38 (R), 13 (R)	447	8.27	<0.0005	48, 16, −10
	Bilateral supplementary motor area, anterior and midcingulate cortex	Bilateral area 24, 32, 6	395	6.68	<0.0005	4, 2, 52
	Fusiform gyrus (L) Inferior occipital gyrus (L)	Area 18 (L), 19 (L)	191	7.31	<0.0005	−28, −74, −20
	Postcentral gyrus (L) Precentral gyrus (L)	Area 3 (L), 4 (L)	107	6.69	<0.0005	−38, −18, 52
	Thalamus (L)	Ventral lateral nucleus (L) Ventral anterior nucleus (L)	80	7.41	<0.0005	−12, −6, 12
	Middle occipital gyrus (R)	-	51	7.29	<0.0005	30, −86, 4
	Fusiform gyrus (R)	-	41	7.35	<0.0005	36, −68, −12
	Intraparietal sulcus (R)	Area 22 (R)	40	6.34	<0.0005	64, −44, 16
	Putamen (R)	-	39	7.10	<0.0005	34, 6, −8
	Insula (L) Putamen (L)	-	35	7.96	<0.0005	−34, −4, −10
	Middle frontal gyrus (R)	Area 10 (R)	32	6.86	<0.0005	26, 54, 18
	Putamen (L)	-	31	6.36	<0.0005	−22, 2, 4
	Inferior orbitofrontal gyrus (L) Insula (L)	-	25	8.15	<0.0005	−40, 20, −8
	Putamen (L)	-	22	6.57	<0.0005	−32, −18, 0
	Inferior parietal gyrus (L)	-	22	6.04	<0.0005	−28, −54, 52
	Inferior occipital gyrus (R)	Area 19 (R)	22	5.70	0.001	44, −82, −10
Baseline > Task	No significant findings					

Overview of significant main task activation clusters. Analyses were FWE-corrected (*p* < 0.05), masked for grey matter, and a minimum cluster size > 20 voxels was used.

**Table 3:** Time-on-task activation clusters within higher difficulty level blocks.

Contrast	Anatomical Regions (AAL Atlas)	Anatomical Regions (Brodmann+ Atlas)	Cluster Size	Peak Z-Value	*p*-Value (Cluster)	x, y, z
Controls > Patients	Bilateral cerebellum 6 Bilateral vermis 6 Bilateral calcarine cortex Bilateral lingual gyrus	Bilateral area 17, 18 23, 30	906	4.04	<0.0005	−2, −74, 0
	Superior temporal gyrus (R) Rolandic operculum (R) Superior temporal pole (R) Insula (R) Inferior orbitofrontal gyrus (R) Inferior frontal operculum (R)	Area 22 (R), 38 (R), 47 (R)	424	4.20	<0.0005	54, 14, −4
	Bilateral medial superior frontal gyrus Bilateral MCC Supplementary motor area (L) Anterior cingulate cortex (L)	Bilateral area 8, 32	260	4.15	<0.0005	0, 24, 52
	Inferior frontal operculum (R)Inferior frontal triangularis (R)	Area 46 (R)	227	4.13	<0.0005	46, 14, 34
	Inferior temporal gyrus (R) Crus Cerebelli I (R) Fusiform gyrus (R) Inferior occipital gyrus (R)	Area 20 (R), 37 (R)	205	4.33	<0.0005	52, −50, −26
	Superior temporal pole (L) Inferior orbitofrontal gyrus (L) Rolandic operculum (L)	Area 22 (L), 38 (L), 47 (L)	190	4.48	<0.0005	−54, 16, −4
	Postcentral gyrus (L) Precentral gyrus (L)	Area 4 (L), 6 (L)	166	3.91	0.001	−42, −16, 50
	Middle temporal gyrus (L)	Area 22 (L)	132	3.86	0.002	−60, −60, 8
	Middle frontal gyrus (L) Precentral gyrus (L)	Area 9 (L)	112	4.23	0.007	−44, 10, 40
	Thalamus (R)	Medial dorsal nucleus (R) Ventral anterior nucleus (R)	106	4.25	0.013	6, −16, 12
	Middle temporal gyrus (L)	Area 22 (L)	106	4.07	0.010	−62, −36, 2
	Inferior occipital gyrus (L) Fusiform gyrus (L) Inferior temporal gyrus (L)	-	105	3.93	0.017	−46, −70, −12
	Angular gyrus (R)	Area 40 (R)	96	4.02	0.024	54, −56, 32
	Middle occipital gyrus (L) Superior occipital gyrus (L)	Area 19 (L)	87	3.99	0.037	−24, −92, 16
	Superior frontal gyrus (L) Middle frontal gyrus (L)	Area 10 (L)	71	3.99	0.017	−30, 62, 16
Patients > Controls	No significant findings					

Significant activation differences between groups in the time-on-task within higher difficulty level blocks. Analyses were cluster-corrected (*p* < 0.05), masked for grey matter, and a minimum cluster size > 20 voxels was used.

**Table 4:** Error-related activation clusters.

Contrast	Anatomical Regions (AAL Atlas)	Anatomical Regions (Brodmann + Atlas)	Cluster Size	Peak Z-Value	*p*-value (Cluster)	x, y, z
Controls > Patients	Precentral gyrus (L) Postcentral gyrus (L)	Area 6 (L)	72	4.22	0.0095	−42, −4, 36
Patients > Controls	No significant findings					

Significant error-related activation differences between groups. Analyses were cluster-corrected (*p* < 0.05), masked for grey matter, and a minimum cluster size > 20 voxels was used.

## Supplementary Materials

The following are available online at https://www.mdpi.com/2076-3425/10/7/419/s1, Supplementary Material 1: Behavioral Analyses.
