# Supplementary material for: The Sustained Attention to Response Task Shows Lower Cingulo-Opercular and Frontoparietal Activity in People with Narcolepsy Type 1: An fMRI Study on the Neural Regulation of Attention
Source: Brain Sci. 2020 Jul 1;10(7):419. doi: 10.3390/brainsci10070419 (PMC7408461; doi:10.3390/brainsci10070419)
Supplement: Supplementary file 1 [file brainsci-10-00419-s001.pdf]

## Supplementary material 1 – Behavioral analyses

**Table S1.** Behavioral performance.

| Difficulty Level | Timeframe | Error Score Patients (%) | | | Error Score Controls (%) | | | *p*-value |
|---|---|---|---|---|---|---|---|---|
| | | Mean | SD | N | Mean | SD | N | |
| Moderate | Overall | 4.43 | 3.73 | 12 | 4.80 | 2.41 | 11 | 0.784 |
| | Repetition 1 | 3.40 | 4.01 | 12 | 3.70 | 3.31 | 11 | - |
| | Repetition 4 | 4.94 | 4.56 | 12 | 7.07 | 3.08 | 11 | - |
| | Early half | 3.77 | 3.27 | 12 | 3.90 | 2.63 | 11 | - |
| | Late half | 5.29 | 5.13 | 12 | 5.77 | 3.44 | 11 | - |
| Higher | Overall | 7.20 | 6.04 | 12 | 5.39 | 2.02 | 11 | 0.347 |
| | Repetition 1 | 8.33 | 8.07 | 12 | 6.76 | 3.21 | 11 | - |
| | Repetition 4 | 6.17 | 5.55 | 12 | 4.71 | 3.35 | 11 | - |
| | Early half | 5.95 | 5.51 | 12 | 3.25 | 2.23 | 11 | - |
| | Late half | 8.33 | 6.37 | 12 | 7.69 | 3.22 | 11 | - |
| **Difficulty Level** | **Timeframe** | **Reaction Time Patients (Ms)** | | | **Reaction Time Controls (ms)** | | | ***p*-value** |
| | | **Mean** | **SD** | **N** | **Mean** | **SD** | **N** | |
| Moderate | Overall | 360.55 | 78.18 | 12 | 313.19 | 30.78 | 11 | 0.074 |
| | Repetition 1 | 362.36 | 101.97 | 12 | 311.79 | 27.51 | 11 | - |
| | Repetition 4 | 363.96 | 78.70 | 12 | 317.29 | 44.07 | 11 | - |
| | Early half | 356.89 | 76.11 | 12 | 315.02 | 25.89 | 11 | - |
| | Late half | 364.87 | 81.56 | 12 | 311.00 | 38.68 | 11 | - |
| Higher | Overall | 362.53 | 75.79 | 12 | 311.06 | 33.59 | 11 | 0.049 |
| | Repetition 1 | 360.78 | 93.35 | 12 | 300.50 | 32.01 | 11 | - |
| | Repetition 4 | 361.06 | 81.24 | 12 | 315.21 | 55.39 | 11 | - |
| | Early half | 368.99 | 87.33 | 12 | 312.80 | 33.02 | 11 | - |
| | Late half | 354.74 | 78.99 | 12 | 308.55 | 36.26 | 11 | - |

**Supplementary S2. Behavioral change from moderate to higher difficulty level.**

| Group | Outcome Measure | Moderate Difficulty | | Higher Difficulty | | *p*-value |
|---|---|---|---|---|---|---|
| | | Mean | SD | Mean | SD | |
| Patients | Error score (%) | 4.43 | 3.73 | 7.20 | 6.04 | 0.011 |
| | Reaction time (ms) | 360.55 | 78.18 | 362.53 | 75.79 | 0.834 |
| Controls | Error score (%) | 4.80 | 2.41 | 5.39 | 2.02 | 0.365 |
| | Reaction time (ms) | 313.19 | 30.78 | 311.06 | 33.59 | 0.361 |

**Supplementary S3. Overall behavioral analyses.**

| Outcome Measure | Source | Sum Of Squares | $Df_1$ | $Df_2$ | Mean Square | F | *p*-value | $\eta_p^2$ |
|---|---|---|---|---|---|---|---|---|
| Error score | Difficulty level | 32.42 | 1 | 21 | 32.42 | 9.04 | 0.007 | 0.30 |
| | Group | 5.89 | 1 | 21 | 5.89 | 0.21 | 0.648 | 0.01 |
| | Difficulty level x Group | 13.50 | 1 | 21 | 13.50 | 3.77 | 0.066 | 0.15 |
| Reaction time | Difficulty level | 0.06 | 1 | 21 | 0.06 | 0.00 | 0.988 | 0.00 |
| | Group | 28033.39 | 1 | 21 | 28033.39 | 4.05 | 0.057 | 0.16 |
| | Difficulty level x Group | 48.37 | 1 | 21 | 48.37 | 0.17 | 0.682 | 0.01 |

**Supplementary S4. Behavioral time-on-task over repetitions analyses.**

| Difficulty Level | Outcome Measure | Source | Sum Of Squares | $Df_1$ | $Df_2$ | Mean Square | F | *p*-value | $\eta_p^2$ |
|---|---|---|---|---|---|---|---|---|---|
| Moderate | Error score | Repetition number | 69.18 | 1 | 21 | 69.18 | 11.88 | 0.002 | 0.36 |
| | | Group | 17.10 | 1 | 21 | 17.10 | 0.74 | 0.401 | 0.03 |
| | | Repetition number x Group | 9.54 | 1 | 21 | 9.54 | 1.64 | 0.214 | 0.07 |
| | Reaction time | Repetition number | 144.54 | 1 | 21 | 144.54 | 0.13 | 0.724 | 0.01 |
| | | Group | 27126.41 | 1 | 21 | 27126.41 | 3.07 | 0.095 | 0.13 |
| | | Repetition number x Group | 43.59 | 1 | 21 | 43.59 | 0.04 | 0.846 | 0.00 |
| Higher | Error score | Repetition number | 50.80 | 1 | 21 | 50.80 | 3.27 | 0.085 | 0.14 |
| | | Group | 26.37 | 1 | 21 | 26.37 | 0.59 | 0.452 | 0.03 |
| | | Repetition number x Group | 0.04 | 1 | 21 | 0.04 | 0.00 | 0.962 | 0.00 |

| | | | | | | | | | |
|---|---|---|---|---|---|---|---|---|---|
| | Reaction time | Repetition number | 644.62 | 1 | 21 | 644.62 | 0.36 | 0.556 | 0.02 |
| | | Group | 32320.55 | 1 | 21 | 32320.55 | 3.96 | 0.060 | 0.16 |
| | | Repetition number x Group | 597.08 | 1 | 21 | 597.08 | 0.33 | 0.571 | 0.02 |

**Supplementary S5. Behavioral time-on-task within blocks analyses.**

| **Difficulty Level** | **Outcome Measure** | **Source** | **Sum Of Squares** | **df$_1$** | **df$_2$** | **Mean square** | **F** | ***p*-value** | $\eta_p^2$ |
|---|---|---|---|---|---|---|---|---|---|
| Moderate | Error score | Block half | 33.03 | 1 | 21 | 33.03 | 4.98 | 0.037 | 0.19 |
| | | Group | 1.07 | 1 | 21 | 1.07 | 0.05 | 0.826 | 0.00 |
| | | Block half x Group | 0.36 | 1 | 21 | 0.36 | 0.05 | 0.818 | 0.00 |
| | Reaction time | Block half | 45.28 | 1 | 21 | 45.28 | 0.13 | 0.723 | 0.01 |
| | | Group | 26301.90 | 1 | 21 | 26301.90 | 3.65 | 0.070 | 0.15 |
| | | Block half x Group | 413.18 | 1 | 21 | 413.18 | 1.18 | 0.290 | 0.05 |
| Higher | Error score | Block half | 133.79 | 1 | 21 | 133.79 | 25.37 | <0.001 | 0.55 |
| | | Group | 32.12 | 1 | 21 | 32.12 | 0.82 | 0.375 | 0.04 |
| | | Block half x Group | 12.23 | 1 | 21 | 12.23 | 2.32 | 0.143 | 0.10 |
| | Reaction time | Block half | 982.51 | 1 | 21 | 982.51 | 0.74 | 0.400 | 0.03 |
| | | Group | 30076.31 | 1 | 21 | 30076.31 | 4.25 | 0.052 | 0.17 |
| | | Block half x Group | 287.70 | 1 | 21 | 287.70 | 0.22 | 0.647 | 0.01 |
